# Spectroscopic and Biological Properties of the 3-Imino-1,8-naphthalimide Derivatives as Fluorophores for Cellular Imaging

**DOI:** 10.3390/molecules28176255

**Published:** 2023-08-25

**Authors:** Mateusz Korzec, Sonia Kotowicz, Katarzyna Malarz, Anna Mrozek-Wilczkiewicz

**Affiliations:** 1 Institute of Chemistry, University of Silesia in Katowice, 9 Szkolna Str., 40-006 Katowice, Poland; sonia.kotowicz@us.edu.pl; 2 August Chelkowski Institute of Physics, University of Silesia in Katowice, 1A 75 Pułku Piechoty Str., 41-500 Chorzow, Poland; katarzyna.malarz@us.edu.pl (K.M.); anna.mrozek-wilczkiewicz@us.edu.pl (A.M.-W.)

**Keywords:** 3-imino-1,8-naphthalimides, cellular imaging, fluorophores

## Abstract

This paper presents the photophysical and biological properties of eight 3-imino-1,8-naphthalimides. The optical properties of the compounds were investigated in the solvents that differed in their polarity (dichloromethane, acetonitrile, and methanol), including three methods of sample preparation using different pre-dissolving solvents such as dimethyl sulfoxide or chloroform. In the course of the research, it was found that there are strong interactions between the tested compounds and DMSO, which was visible as a change in the maximum emission band (λ_em_) of the neat 3-imino-1,8-naphthalimides (λ_em_ = 470–480 nm) and between the compounds and DMSO (λ_em_ = 504–514 nm). The shift of the emission maximum that was associated with the presence of a small amount of DMSO in the sample was as much as 41 nm. In addition, the susceptibility of imines to hydrolysis in the methanol/water mixture with increasing water content and in the methanol/water mixture (*v*/*v*; 1:1) in the pH range from 1 to 12 was discussed. The studies showed that the compounds are hydrolysed in the CH_3_OH/H_2_O system in an acidic environment (pH in the range of 1 to 4). In addition, it was found that partial hydrolysis occurs in systems with an increased amount of water, and its degree may depend on the type of substituent on the imine bond. The compounds tended to quench the emission (ACQ) in the aggregated state and increase the emission related to the protonation of the imine bond. Moreover, it was found that the substituent in the imine bonds influenced a compound’s individual photophysical properties. Biological tests, including cytotoxicity studies and cellular localisation, were also performed for all of the molecules. All of the tested compounds exhibited green fluorescence in the MCF-7 cells and showed co-localisation in the mitochondria, endoplasmic reticulum, and lysosome. The obtained photophysical and biological results indicate the promising potential use of the tested compounds as cellular dyes.

## 1. Introduction

**Figure 1:**
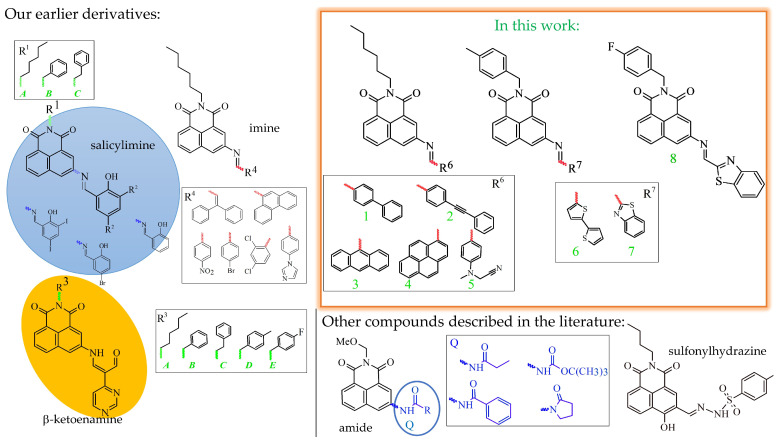
Examples of 3-substituted-1,8-naphthalimide derivatives for cellular imaging and the structure of compounds described in this work (ImNIDs 1–8).

Compounds that contain the 1,8-naphthalimide units have shown many essential properties for their further use. The literature has mainly described the 4C-substituted 1,8-naphthalimide derivatives that have been used in organic electronics [1,2], for detecting ions [3], as biologically active compounds [4], and for cellular imaging [3,5]. However, to the best of our knowledge, suitable imines substituted at the 3C-position of the naphthalene ring in the naphthalimide structure have yet to be synthesised. Thus, despite the extensive research into 1,8-naphthalimide described in the 4C-position, the *N*-substituted derivatives mainly include secondary or tertiary amines, amides, and salicyl-imines. The 1,8-naphthalimides with secondary or tertiary amines were investigated as ion sensors, e.g., Pb^2+^ [6], Zn^2+^ [7], fluorophores for cellular imaging [8] or for imaging the subcellular compartments in plants [9], for imaging the ions in living cells such as Zn^2+^ [10], or DNA binding [11,12], as hypoxia sensors [13], and their optical properties have also been investigated [14,15,16,17,18]. In addition, several reports have included derivatives with a substituted hydroxyl group at the 2-position of the imine bond (salicyl-imines). These compounds were used to detect CN^−^, F^−^, and Al^3+^, which play important roles in organisms [15,16,17,18,19,20,21]. Compounds substituted at the 4-position of the ring that were used for cellular imaging are presented in the review paper [5]. There have been a few reports of derivatives substituted at the 3-C position for use in cellular imaging. These include derivatives that have been substituted with groups (Figure 1): amide [22] or sulfonylhydrazine [23]. In addition, it is noteworthy that 3,4-disubstituted 1,8-naphthalimide has also been used in cellular imaging [24,25].

However, the first information on *N*-substitutes at the 3C-position of 1,8-naphthalimides, e.g., β-ketoenamine, salicyl-imine, and imine, was provided in our earlier works (Figure 1) [26,27,28,29]. As a result, knowledge of the properties of *N*-substituted-1,8-naphthalimides is gradually expanding. Our previous work with *N*-substituted-1,8-naphthalimides revealed some interesting photophysical properties of these compounds, which, combined with their good permeability in the cellular environment, enable their application in the field of cell bioimaging [28,29,30]. Generally, fluorescent compounds can be used for various types of cellular staining, biological processes, cell state monitoring, detecting metal ions, histopathology, and even clinical diagnostics. Therefore, they must have several distinctive characteristics to become valuable dyes. The first crucial parameter is a strong fluorescence with a high quantum yield to reduce interference from any intracellular autofluorescence and excitation light sources. In addition, an absorption of more than 340 nm increases the signal-to-noise ratio and significantly influences the quality of the desired images. Another crucial application criterion for potential dyes is photostability and low photobleaching, which guarantee signal stability during prolonged cell staining [31,32,33]. One more characteristic of a good cellular dye is that it does not exhibit toxicity. This characteristic enables long-term incubation for fixed preparations [34,35].

This paper presents previously undescribed photophysical and biological research for the 3-imino-1,8-naphthalimide series (ImNIDs). The presented compounds are derivatives of 1,8-naphthalimide with some modifications, including various substituents in the imide part (substituents: hexyl, fluorobenzyl, methylbenzyl) and in the imine part (phenyl and biphenyl, bitiophenes, benzothiazoles, or fused rings: anthracene, pyrene). The optical studies included investigations conducted in various solvents (dichloromethane, acetonitrile, and methanol), the aggregation-induced emission (AIE) in the CH_3_OH/H_2_O system with an increase of the water fraction, the protonation of the imine bond in acetonitrile and methanol, as well as the properties of compounds in a mixture of CH_3_OH/H_2_O (*v*/*v*; 1:1) at pH in the range of 1 to 12. Moreover, the influence of the chemical structure of the compounds on their photophysical properties was also analysed. The biological studies concerned the determination of the cytotoxicity of the compounds as well as the cell staining. Moreover, the co-localisation of these compounds was determined by the example of compound **5** with a (2-cyanoethyl)methylamine) unit.

## 2. Results and Discussion 

### 2.1. Characteristics of 3-Imino-1,8-naphthalimide Derivatives

1,8-Naphthalimide derivatives with imine linkages, which are denoted as ImNIDs, were obtained in a three-step reaction that involved condensing the anhydride with an amine, reducing the -NO_2_ to -NH_2_, and then condensing with the appropriate aldehydes [26,27,28]. In Figure 1, the structures of the analysed imines are presented. The ImNIDs **1**–**5** were characterised in our previous work [36], while the ^1^H and ^13^C NMR spectra and elemental analysis for imines **6**, **7**, and **8** are presented in the Supplementary Materials (ESI).

### 2.2. Optical Properties

Optical investigations of the ImNIDs were performed in solvents that differed in their polarities, such as dichloromethane (CH_2_Cl_2_), methanol (CH_3_OH), and acetonitrile (CH_3_CN). The research included using various methods to prepare the sample and analysing the obtained results. Moreover, the ability to initiate aggregation-induced emission was also studied, and any changes in the properties due to the protonation of the imine bond were demonstrated.

#### 2.2.1. Absorption and Emission Investigations

**Figure 2:**
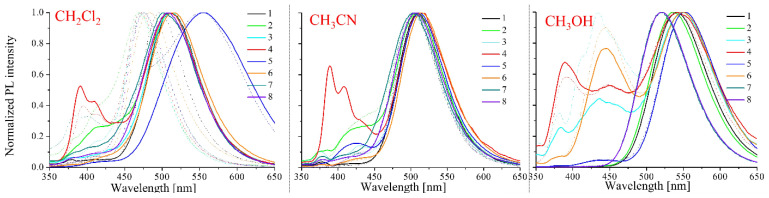
The emission spectra of the compounds that had been dissolved in CHCl_3_ (dashed line) or DMSO (solid line) and then diluted in various solvents (dichloromethane, acetonitrile, and methanol) that were obtained at the excitation wavelength (λ_ex_ = 340 nm).

An optical study was performed in solvents that had different polarities, such as CH_2_Cl_2_, CH_3_OH, and CH_3_CN. The research consisted of using three different methods to prepare the samples: directly in the solvent (0.1 mM), pre-dissolved in DMSO (1 mM), or CHCl_3_ (1 mM). The samples were then diluted to a concentration of 10 μM in the tested solvent solutions (CH_2_Cl_2_, CH_3_OH, and CH_3_CN). In some cases, preparing the samples directly in solvents with a concentration of 0.1 mM was impossible. All of the compounds were soluble in dichloromethane; only compound **4** was insoluble in acetonitrile; and most compounds were insoluble in methanol (ImNDIs: **3**, **4**, **6**, **7**, and **8**) (Table 1). On the other hand, preparing samples with a concentration of 1 mM pre-dissolved in DMSO or chloroform was relatively easy. These samples were then diluted in the test solvent to a concentration of 10 μM. Moreover, photoluminescence (PL) quantum yield (Φ) measurements were performed for the compounds that had been dissolved in the dichloromethane and methanol solutions. The results are presented in Table 1, Figure 2 and Figure S2.

**Figure 3:**
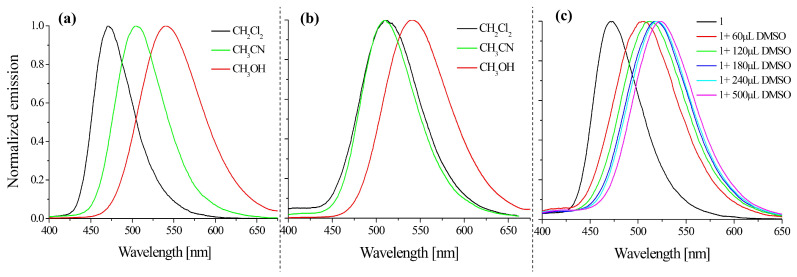
The emission spectra of compound **1**, which had been pre-dissolved in CHCl_3_ (**a**) or DMSO (**b**) and then diluted in the solvents: dichloromethane, acetonitrile, and methanol (λ_ex_ = 340 nm). (**c**) The emission spectra of compound **1**, which had been dissolved in CHCl_3_ and then diluted in dichloromethane with the addition of DMSO (λ_ex_ = 340 nm).

The maximum fluorescence emission band (λ_em_) in dichloromethane that was obtained by dissolution in the solvent or pre-dissolved in chloroform was within the range of 470 to 484 nm, except for compound **5** (containing a 2-cyanoethyl)methylamine substituent) with λ_em_ = 553 nm and compound **4** (containing pyrene) with λ_em_ = 393 nm, whereas the maximum emission band was in the range of 504 to 512 nm in acetonitrile. In the case of methanol, the position of the λ_em_ range was from 520 to 550 nm (cf. Table 1, Figure 2). Therefore, a bathochromic shift of the emission maximum with increasing solvent polarity can be considered (Table 1 and Figure 3a). For compound **4** (containing pyrene), the emission was in a double range: a vibrionic structure band from 350 to 450 and an intense band from 450 to 600 nm, which is characteristic for derivatives with this substituent. The first band is typical for monomeric fluorescence emission, whereas the second emission band is associated with excimer-forming behaviour [37,38]. The visible changes in the form of the two emission ranges for the compounds **2**, **4**, **5,** and **6** might also be related to the formation of excimers, as has been reported in the literature [39].

The influence of a substituent in an imine bond was also visible in the quantum yield. In dichloromethane, most of the compounds were characterised by a low quantum yield (0.1% to 1.9%) regardless of sample preparation, although compound **5** had a quantum yield of more than 10%. In turn, the lowest quantum yield in methanol was found in compounds with a pyrene substituent (**4**) and a thiophene substituent (**6**), whereas the compounds that contained the benzothiazole substituent (**7** and **8**) had a significant increase in the quantum yield (12.34% to 19.24%). Compound **5** in methanol had a lower quantum yield than the one in dichloromethane. For the remaining compounds (**1**, **2**, and **3**), the quantum yield increased slightly relative to dichloromethane.

It was observed that the solvent polarity or its ability to form hydrogen bonds significantly affected the optical properties (mainly the emissions) of the analysed compounds. The changes in the position of the absorption band maxima were insignificant, while the emission started to change. There were no noticeable changes in the absorption spectra (Table 1). However, there were differences in the obtained emission spectra of the compounds in a slightly polar solvent (CH_2_Cl_2_) (cf. Table 1, Figure 2 and Figure S2). Pre-dissolving the compounds in DMSO and then diluting them in a slightly polar solvent (dichloromethane) produced a different emission maximum than in the samples that were obtained from those that had been directly dissolved in the test solvent. 

The properties of all of the compounds in acetonitrile and methanol were not characterised by any significant differences that were dependent on the sample preparation method. The differences in emissions that were dependent on the sample preparation method in the tested solvents were as follows: in dichloromethane, 30 to 41 nm (except compound **5**); in acetonitrile, 0 to 7 nm; and in methanol, 0 to 5 nm (Table 1, Figure 2 and Figure S2). Based on these results, it can be concluded that intermolecular interactions form between the imine and DMSO, which is discussed in the next part of the paper.

Thus, the influence of the polarity of the solvent on the emission change was visible (Figure 3), which proved that there was an interaction between the tested compounds and the solvent (solvatochronism). Our interest was aroused by the change in emission of all compounds that were initially dissolved in DMSO and then diluted in dichloromethane. For example, the emission of compound **1** in dichloromethane was at 470 nm, while in the DMSO system it was at 509 nm, which was a change of λ_max_ at 39 nm (Table 1, Figure 3a,b). In the next experiment, the ImNIDs were pre-dissolved in CHCl_3_ and then diluted in dichloromethane with the appropriate DMSO content. The collected spectra are presented in Figure 3c. It can be seen that with an increase in the amount of DMSO in the system, the emission band shifted bathochromically. This demonstrates the strong effect of DMSO on 1,8-naphthalimides that was observed in this experiment. The experiments also showed that the interactions between the ImNIDs and the presence of a polar compound (DMSO) affected the emission properties. Based on the above considerations, it can be concluded that the ability of 1,8-naphthalimide derivatives to form intermolecular interactions might significantly affect the photophysical properties of the system, which has also been considered in other works [40,41].

#### 2.2.2. Optical Properties of the Aggregates

**Figure 4:**
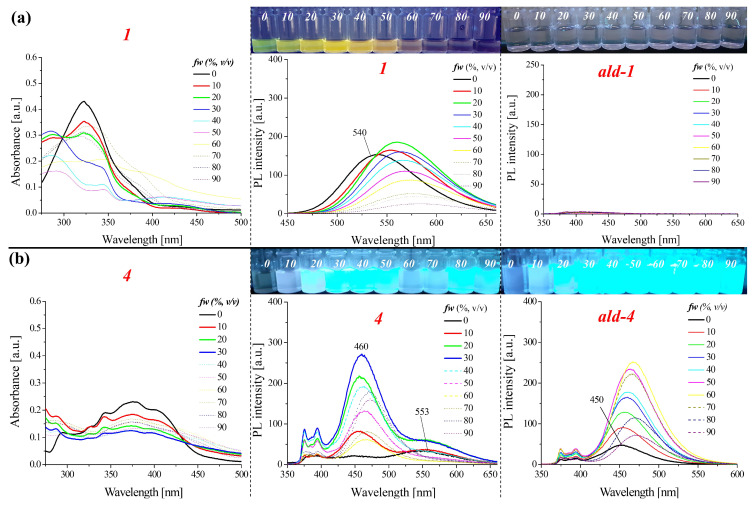
Absorption and emission properties in a binary mixture of CH_3_OH/H_2_O with an increasing water content (*fw*) for: (**a**) 1 and *ald-1*; (**b**) 4 and *ald-4*. The photographs were taken under 365 nm UV irradiation from a hand-held UV lamp.

Studies of the 1,8-naphthalimide derivatives PL properties in the CH_3_OH/H_2_O mixture with a different water content in the system were also performed. The PL spectra were measured for excitation λ_ex_ = 340 nm two hours after the solution had been prepared. The results are presented in Figure 4, Figures S3 and S4. The capacity of the compounds for aggregation-induced emission (AIE-gens) affects their potential use [42]; therefore, this type of research provides some information about the nature of the compounds. Most importantly, a slight change in the structure of a compound might favourably affect the properties that are displayed in the aggregated state [43]. Our previous research, in which naphtalhimides with imine bonds were compared with β-ketoenamines, also showed this [27]. In the case of azomethines, the ability of the compounds to hydrolyse is also taken into account [44], which makes the analysis of the properties of these derivatives more difficult. All of the compounds exhibited the aggregation-caused quenching (ACQ) emission mechanism, except for compound **4** (cf. Figure 4, Figures S3 and S4). However, unlike the molecules that had been described earlier [28,29], the influence of the substituent on the range of the emitted light that was caused by the aggregation was observed. Changes in the tested system for compounds **4** and **6** occurred in the range of blue light, while for compounds **7** and **8**, they occurred in the range of green light. Furthermore, the hydrolysis process determines the photophysical or biological properties of the imines [28,44]. For this reason and in order to perform a better analysis, CH_3_OH/H_2_O mixture tests were also conducted for the appropriate aldehydes. However, the emission measurement was performed with an excitation that corresponded to the maximum absorption band in methanol (Figure 4 and Figure S5b). The studies showed that the four aldehydes (4-(phenylethynyl)benzaldehyde (*ald-2*), 9-antracenecarboxaldehyde *(ald-3*), 1-pyrenecarboxaldehyde (*ald-4*), and 2,2′-bithiophene-5-carboxaldehyde (*ald-6*)) exhibited the ability to AIE. Other substrates, i.e., biphenyl-4-carboxaldehyde (*ald-1*), 4-[(2-cyanoethyl)methylamino]benzaldehyde (*ald-5*), and benzothiazole-2-carboxaldehyde (*ald-7*), do not show fluorogenic properties (Figure 4b and Figure S5a). In the case of compounds **2** and **4**, it was observed that with a higher water content in the system, an emission band of the corresponding aldehyde was observed (compare Figure 4, Figures S3 and S4). This might indicate the partial hydrolysis of the tested ImNIDs and the aggregation of the fluorogenic aldehyde, as has also been reported by other researchers [45]. However, in the case of compounds **4**, **6**, **7,** and **8**, the visible effect that was caused by the aggregation was different than for the other tested ImNIDs (**1**, **3**, and **5**) (Figure 4, Figures S3 and S4).

#### 2.2.3. Protonation of the Imine Bond

As has been shown in a previous study, forming an imine bond at the 3-position of the naphthalimide naphthalene ring causes a decrease in the quantum efficiency relative to the substrate (amine). The fluorescence quantum yield for the amine was 38–86%, while for the corresponding imine, it was in the range of 1–26% depending on the solvent [28]. Moreover, the compounds presented in this work were characterised by a low fluorescence quantum yield in the range of 0.1 to 19%, depending on the solvent (Table 1). The low fluorescence quantum yield for the analysed ImNIDs might be due to a photoinduced electron transfer (PET on) or might have prevented the internal charge transfer (ICT off) due to the presence of an imine bridge. The emission mechanisms associated with the occurrence of ICT and PET on naphthalimide derivatives have been analysed in the literature. It should be emphasised that the ICT effect from item 3-C is weaker than from item 4-C [46]. In addition, considerations based on the available literature determine that the fluorescence quenching by photoinduced electron transfer for naphthalimide derivatives is accelerated in the “lower-receptor” systems, i.e., those substituted in the naphthalene ring [47]. In addition, other studies have shown that the introduction of a methoxy group to the compound reduced the quantum efficiency, which was caused by intramolecular photoinduced electron transfer (PET) [48].

**Figure 5:**
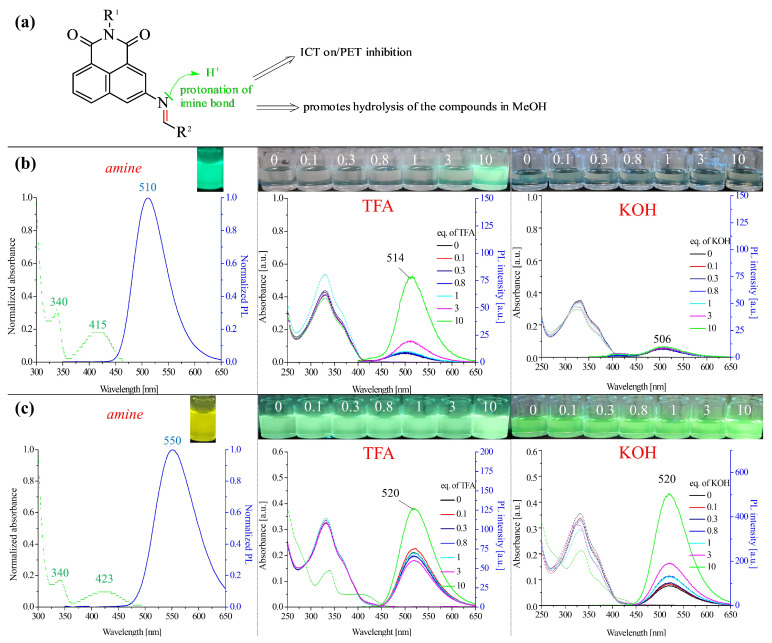
(**a**) Scheme of the protonation of an imine bond; optical properties of the substrate (amine) in the presence of TFA (10 eq.) and the effect of the TFA and KOH concentrations on the photoluminescence properties of compound **7** in (**b**) acetonitrile and (**c**) methanol. The photographs were taken under 366 nm UV irradiation from a hand-held UV lamp.

In this work, the protonation of the imine bond, which should result in an increase in the emission intensity of the tested compound, was investigated. The investigation in this field was performed in two solvents (CH_3_CN and CH_3_OH), and the results are presented in Figure 5, Figures S5 and S6. The concentration of the compounds was 10 μm, while the TFA concentration range was 1–100 μm (0.1–10 eq). 

In order to thoroughly analyse the effect of TFA on the tested imine, the possibility of PET inhibition, on the one hand, and the occurrence of the compound hydrolysis process, on the other hand, should be considered (Figure 5a). For this purpose, the substrate (amine) was tested in the analysed solvents at 10 eq. TFA, and the results are summarised in Figure 5b. In previous studies, a negligible effect of the TFA concentration on the properties of amine in each solvent was shown [29]. Therefore, in this work, the analysis of the amine properties was performed with one TFA concentration. In the case of the remaining compounds, the amount of TFA increased (Figure 5c, Figures S6 and S7). Based on the obtained results, it can be concluded that both the type of solvent and the substituent at the imine bond affect the protonation of the imine bond and further possible transformations (Figure 5a).

In methanol, visible changes in the absorption and emission spectrum occurred at 10 eq. TFA (Figure S6). Interestingly, for most of the compounds in CH_3_OH, the obtained spectra corresponded precisely to the amine (compare Figure 5c and Figure S6). It can therefore be assumed that the hydrolysis of the analysed compounds takes place in this environment, except for compound **7** (with the benzothiazole substituents). A clear shift of the emission maximum from about 540 nm to 550 nm was observed, similar to those in the case of compounds **1** and **2** (with biphenyl substituents), or a significant increase in emission at the maximum of 550 nm for compounds **3**, **4**, **5,** and **6**. In acetonitrile, a negligible effect of the solvent on the hydrolysis of the compounds was observed, except for compound **1**. Thus, the increase in emission in this solvent might be related to the imine bond’s protonation, which favours PET inhibition (Figure S7).

#### 2.2.4. Properties of Compounds at Different pHs

**Figure 6:**
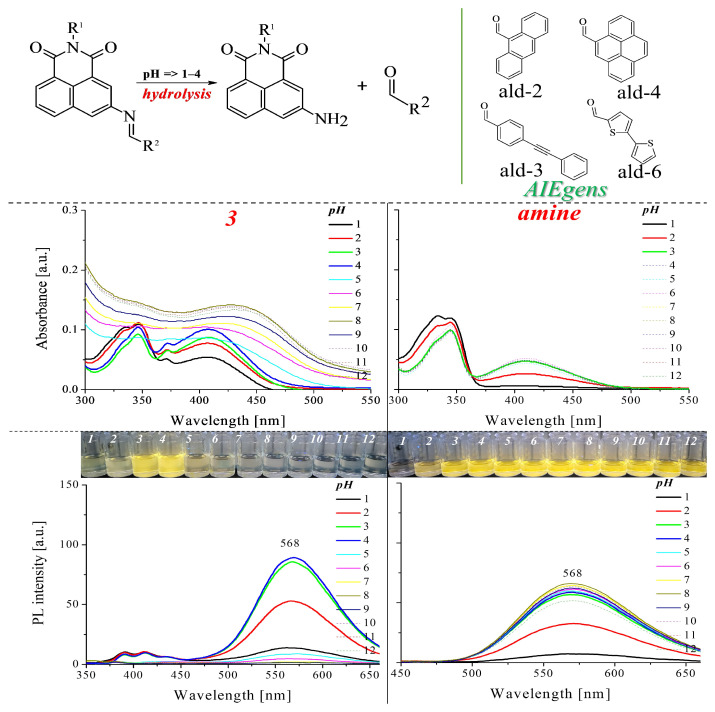
Optical properties of compounds at different pHs (1 to 12) when mixing CH_3_OH and H_2_O in a 1:1 volume ratio. The concentration of the compound is 10 μM. Photographs were taken under 366 nm UV irradiation from a hand-held UV lamp.

The tests were conducted in a mixture of CH_3_OH and H_2_O with a volume ratio of 1:1 in the pH range of 1 to 12. The pH of the mixture was adjusted with 1 or 0.1 M HCl and KOH using a pH meter. The results of the UV and PL measurements are gathered in Figure 6 and Figure S8.

Analysis of the obtained results in a mixture with a pH of 1 to 4 proves the hydrolysis of imines. Therefore, in this pH range (from 1 to 4), the absorption spectra should be attributed to the substrates obtained. As demonstrated, the properties of the amine (3-amino-1,8-naphthalimide) in this mixture at pH in the range of 3 to 12 are not changed. The amine can be protonated at pH 1 and 2, which quenches the emission. This can be confirmed by the absence of a visible UV band in the range of 360–500 nm and lower emission intensity at these pH values (Figure 6). The properties of the appropriate aldehydes are shown in Figure S5. For two imines (4, 6) in mixtures at pH 1 to 4, the effect obtained was related to the aggregation of the aldehyde formed from the hydrolysis of the compounds. On the other hand, for compounds **2** and **3**, the emission from both the amine and the aldehyde is visible in the spectra (compare Figures S5 and S8). Whereas, for compounds **1** and **7**, the emission from the amine is visible. The spectra above pH 5 show partial aggregation of compounds and quenching of emissions (compared with Figures S3 and S4), which may mean that the hydrolysis of compounds in this system does not occur. The exception is compound **7**, which, at pH above 11, shows changes indicative of its hydrolysis. In the presence of about 10 KOH equivalents, it should be emphasised that this compound showed changes in the UV spectrum and increased emission in methanol, as shown in Figure 5.

### 2.3. Biological Studies: Cytotoxicity and Cellular Imaging

The first step in the study to determine the suitability of the tested compounds as dyes was to check their toxicity. For this purpose, several lines of common cancer, including colon (HCT 116), breast (MCF-7), and glioblastoma (U-251), as well as normal (NHDF) cells, were selected. The results, which are presented in Table 2, proved that most of the tested 1,8-naphthalimide derivatives did not exhibit any significant toxicity to the tested cell lines when they had been incubated with them for 72 h. The exceptions were one N-hexyl-1,8-naphthalimide compound **3** and two compounds (**7** and **8**) that contained the benzothiazole substituent, which exhibited a slight cytotoxicity against the HCT 116 and MCF-7 cell lines. However, it should be noted that these compounds are suitable for short-term staining. Notably, all of the tested compounds were able to reach maximum fluorescence in the cells 1–2 h after administration. This property, combined with the interesting spectroscopic features, was the reason for further studies involving cellular localisation.

**Figure 7:**
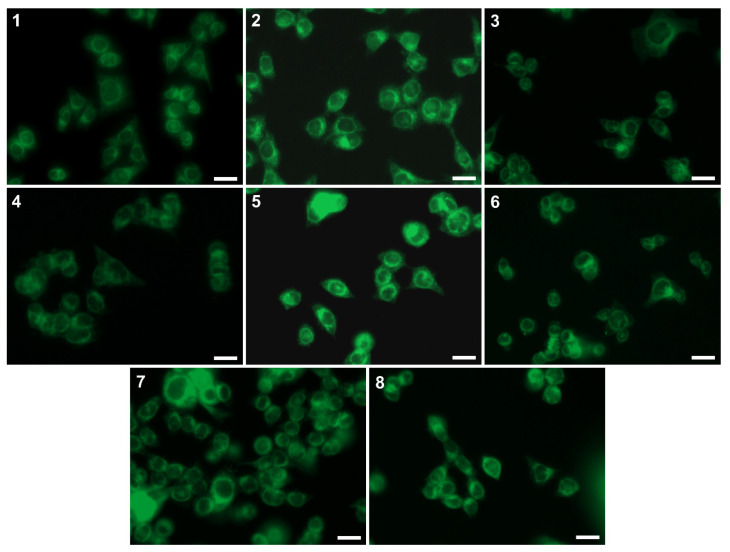
The MCF-7 cells had been stained with tested compounds (**1**–**8**) at 25 µM for 2 h. The images were acquired under 470 nm LED illumination. Scale bars indicate 25 µm.

Based on previous spectroscopic observations, a series of cell staining experiments with all of the tested compounds were conducted. The absorption profiles of the tested derivatives indicated that they could be excited under LED illumination at two wavelengths, 365 nm and 470 nm. It is worth noting that excitation in the longer wavelength range, which reduces interference and noise from the background and autofluorescence of intracellular components, can be particularly valuable. As shown in Figure 7, all of the tested compounds exhibited a green fluorescence in the MCF-7 cells. The signal was sufficient in all cases, with no signs of any rapid photobleaching. 

The images indicated good cellular penetration of the tested compounds, with a clear localisation within the membranous structures in cells. Moreover, it was observed that compounds **5**–**8** exhibited stronger fluorescence and better behaviour in the cellular environment than the others. This may be related to the lipophilicity of these compounds, which ranges from 4.03 to 5.70 (LogP values presented in Table 2). Finally, we performed co-localisation studies with commercial specific-organelle dyes, including MitoTracker, ERTracker, and LysoTracker. After analysing the spectroscopic data, we selected derivative **5** for further experiments due to the shift of the emission maximum towards the longer wavelengths. Dyes with an emission spectrum that is shifted towards a longer wavelength, such as around 550 nm-emitted green light, are more desirable and are one of the most widely used in the wide-ranging research related to cell imaging.

**Figure 8:**
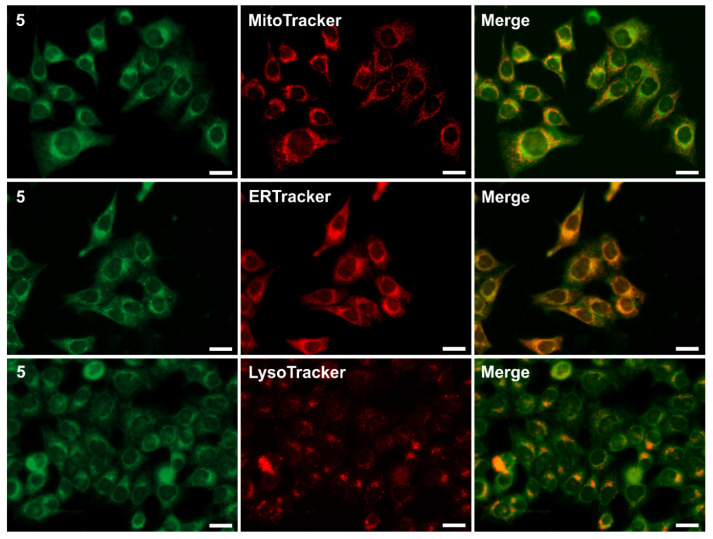
Co-localisation studies of compound **5** with organelle dyes (MitoTracker, ERTracker, and LysoTracker) in the MCF-7 cells. Scale bars indicate 25 µm.

Co-localisation studies of imino-1,8-naphthalimide **5** with organelle dyes dedicated to the mitochondria, endoplasmic reticulum, or lysosomes are presented in Figure 8. The overlapped images indicated that compound **5** penetrated the outer cell membrane and tended to have a non-selective accumulation in the membranous organelles. These data are consistent with our previous results for N-hexyl-1,8-naphthalimides and 3-imino-(2-phenol)-1,8-naphthalimides, which preferentially localise in the mitochondria, endoplasmic reticulum, and lysosomes [28,29]. This landscape of localisation of the tested compounds might also be influenced by their slight lipophilic nature. In addition, the visualisation results were confirmed by calculating the Pearson’s correlation coefficient (PCC). The Pearson’s correlation coefficient for the accumulation of compound **5** in all tested organelles was calculated in ImageJ software (ver. 1.41). The scatter plots of co-localisation are depicted in Figure S9. As is presented in Table 3, the PCC had high values (above 0.776) for all of the stained organelles. Moreover, it should be emphasised that to date the most promising in cellular imaging have been 3-imino-1,8-naphthalimides (salicylic derivatives) substituted with n-hexylamine, whereas in the case of other substituents the effect was weaker [28,41]. In this work, we showed that the compounds with benzyl substituents (**6**–**8**) were also suitable for cellular staining (Figure 7).

## 3. Conclusions

The paper presents optical and biological investigations of eight imino-1,8-naphthalimide derivatives. During the research, there was a strong interaction of DMSO with the tested compounds, which affected the emission properties of the solvents. Moreover, the influence of the compound structure on the optical properties was analysed and showed that benzothioazole-substituted derivatives have a high quantum yield in methanol (Φ = 12.34–19.24%). The experiment when trifluoroacetic acid (TFA) was added showed an increase in the intensity of the emission (PL), which was related to the inhibition of the PET process. The aggregation research (CH_3_OH/H_2_O) showed that the compounds are characterised by the quenching of the emission that is caused by the aggregation (ACQ), except for compound **4**, which had a pyrene substituent. In the tests carried out in the CH_3_OH/H_2_O system at different pHs in the range from 1 to 12, it was found that all compounds in the system with pHs 1 to 4 are subject to hydrolysis. In the case of two compounds (**2** and **4**), the presence of the starting aldehyde was noticeable, which proved the partial hydrolysis of the analysed compounds. Since the compounds exhibited no biological activity, cellular imaging studies were performed on them, which had a better staining effect in the case of compounds **5**–**8** than for the other compounds. Based on the colonisation studies, the placement of compounds in the mitochondria and the intraplasmic reticulum was determined. Interestingly, not only the compounds with n-hexylamine were suitable for cellular imaging but also those with benzylic substitutions, which was shown in this work for the first time.

## Figures and Tables

**Table 1:** The spectroscopy data of the imino-1,8-naphtalimides in various solvents (c = 10 μM).

Medium	dis. in:	Code	λ_max_ (ε × 10^4^)	λ_em_	Stokes Shift	Φ	Code	λ_max_ (ε × 10^4^)	λ_em_	Stokes Shift	Φ
			[nm]	[nm]	[nm]	[%]		[nm]	[nm]	[nm]	[%]
CH_2_Cl_2_	solvent	**1**	324^4.62^, 372^sh^	470	146	-	**2**	334^7.21^, 374^sh^	470	136	-
	CHCl_3_ ^a^		330^2.87^, 372^sh^	470	140	1.65		335^4.01^, 372^sh^	470	135	0.1
	DMSO ^a^		325^4.8^, 372^sh^	509	184	0.97		335^6.76^, 372^sh^	511	176	0.16
CH_3_CN	solvent		320^5.74^, 373^sh^	504	184	-		330^14.85^, 372^sh^	508	178	-
	CHCl_3_ ^a^		330^4,5^, 373^sh^	504	174	-		330^5.16^, 370^sh^	501	171	-
	DMSO ^a^		320^4,5^, 372^sh^	509	189	-		330^6.52^, 370^sh^	508	178	-
CH_3_OH	solvent		324^5.65^, 374^sh^	540	216	-		333^8.13^, 430^sh^	538	205	-
	CHCl_3_ ^a^		324^2.9^, 371^sh^	540	216	4.7		333^4.13^, 427^sh^	538	205	4.95
	DMSO ^a^		325^3.6^, 370^sh^	540	215	5.25		332^5.40^, 427^sh^	538	206	4.94
CH_2_Cl_2_	solvent	**3**	335^1.22^, 411^1.48^	473	62	-	**4**	380^4.43^, 406^4.01^	472	66	-
	CHCl_3_ ^a^		321^1.01^, 415^1.26^	473	58	1.9		379^2.14^, 409^1.92^	472	63	0.47
	DMSO ^a^		337^1.55^, 410^1.88^	511	101	0.28		379^3.49^, 408^3.18^	520	112	0.96
CH_3_CN	solvent		337^2.06^, 406^2.39^	505	99	-		-	-	-	-
	CHCl_3_ ^a^		336^1.37^, 406^1.59^	505	99	-		375^2.93^, 403^2.51^	512	109	-
	DMSO ^a^		335^1.54^, 407^1.75^	507	100	-		376^3.72^, 405^3.12^	512	107	-
CH_3_OH	solvent		-	-	-	-		-	-	-	-
	CHCl_3_ ^a^		338^1.75^, 410^1.59^	537	127	3.92		342^2.32^, 375^2.39^, 407^2.05^	548	141	0.15
	DMSO ^a^		330^1.75^, 410^1.94^	537	127	0.36		344^2.12^, 376^2.26^, 408^1.94^	553	145	0.19
CH_2_Cl_2_	solvent	**5**	350^6.78^	470, 553	120	-	**6**	350^4.29^, 384^4.85^	475	91	-
	CHCl_3_ ^a^		348^2.26^	480, 553	132	10.83		350^2.25^, 382^2.57^	484	102	1.26
	DMSO ^a^		350^3.97^	557	207	11.32		350^3.11^, 384^3.43^	514	130	0.39
CH_3_CN	solvent		348^3.37^	507	159	-		350^4.68^, 377^5.13^	510	133	-
	CHCl_3_ ^a^		347^2.72^	507	160	-		347^3.28^, 380^3.53^	510	130	-
	DMSO ^a^		347^4.23^	511	164	-		347^3.31^, 380^3.56^	514	134	-
CH_3_OH	solvent		347^4.27^	550	203	-		-	-	-	-
	CHCl_3_ ^a^		347^2.43^	545	198	2.37		350^3.04^, 377^3.27^	443, 550	66	1.35
	DMSO ^a^		346^3.17^	550	204	3.69		350^3.14^, 377^3.43^	440, 550	63	1.34
CH_2_Cl_2_	solvent	**7**	333^5.77^, 375^sh^	473	140	-	**8**	333^4.90^, 374^sh^	476	143	-
	CHCl_3_ ^a^		333^2.67^, 375^sh^	474	141	0.15		332^4.42^, 375^sh^	475	143	0.91
	DMSO ^a^		333^3.66^, 375^sh^	504	171	0.27		333^4.08^, 375^sh^	508	175	0.76
CH_3_CN	solvent		329^6.20^, 368^sh^	503	174	-		330^6.50^, 370^sh^	502	172	-
	CHCl_3_ ^a^		332^2.99^, 369^sh^	504	172	-		330^5.40^, 370^sh^	502	172	-
	DMSO ^a^		331^3.59^, 369^sh^	504	173	-		330^4.21^, 370^sh^	506	176	-
CH_3_OH	solvent		-	-	-	-		-	-	-	-
	CHCl_3_ ^a^		332^2.66^, 375^sh^	520	188	17.64		332^4.64^, 375^sh^	520	188	19.24
	DMSO ^a^		331^2.99^, 375^sh^	520	189	12.34		332^3.34^, 375^sh^	520	188	15.38

^a^ Concentration of compounds in the tested solvents (CH_2_Cl_2_, CH_3_OH, and CH_3_CN) => c = 10 mM; ε—absorption coefficient, [dm^3^·mol^−1^·cm^−1^]; ^sh^—shoulder; Stokes shifts calculated according to the equation Δλ = (λ_em_ − λ_abs_) [nm].

**Table 2:** Cytoxicity studies of iminonaphtalimides on cancer and normal cell lines were evaluated.

Code		Antiproliferative Activity–IC_50_ [µM]			
	LogP *	HCT 116	MCF-7	U-251	NHDF
**1**	6.17 ± 0.91	>25	>25	>25	>25
**2**	7.08 ± 0.94	>25	>25	>25	>25
**3**	6.98 ± 0.90	22.27 ± 2.12	21.31 ± 1.64	>25	>25
**4**	7.13 ± 0.93	>25	>25	>25	>25
**5**	4.03 ± 0.92	>25	>25	>25	>25
**6**	5.39 ± 0.96	>25	>25	>25	>25
**7**	5.70 ± 0.96	20.31 ± 2.36	17.90 ± 1.36	>25	>25
**8**	5.29 ± 1.00	24.23 ± 3.26	>25	>25	>25

* LogP is calculated in the ChemSketch programme.

**Table 3:** Pearson’s correlation coefficient calculated for mitochondria, endoplasmic reticulum, and lysosomes.

Code	Pearson’s Coefficient		
	Mitochondria	Endoplasmic Reticulum	Lysosomes
**5**	0.852	0.943	0.776

## Data Availability

The data are available in this publication and Supplementary Information.

## Supplementary Materials

The following supporting information can be downloaded at: https://www.mdpi.com/article/10.3390/molecules28176255/s1. Figure S1: ^1^H NMR in DMSO-*d_6_* for compounds **1**–**5** and in CDCl_3_ for compounds **6**–**8**; Figure S2: Emission spectra of the tested compounds depending on the sample preparation method; Figure S3: Absorbance and photoluminescence (PL) properties in a binary mixture of MeOH/H_2_O with an increasing water content (*fw*) for imines (2,3,5); Figure S4: Absorbance and photoluminescence (PL) properties in a binary mixture of MeOH/H_2_O with an increasing water content (*fw*) for imines (6,7,8); Figure S5: (a) The photoluminescence (PL) properties in a binary mixture of MeOH/H_2_O with an increasing water content (*fw*) for the corresponding aldehydes (ald-2,3,5,6,7). Photographs were taken under 365 nm UV irradiation from a hand-held UV lamp; (b) UV-VIS spectra for tested aldehydes in methanol at a concentration of 10 μM; and (c) absorption and emission properties in a binary mixture of MeOH/H_2_O with an increasing water content (*fw*) for the 3-amino-N-hexyl-1,8-naphthalimide;Figure S6: Effect of TFA on photoluminescence (PL) properties of imines in methanol; Figure S7: Effect of TFA on photoluminescence (PL) properties of imines in acetonitrile; Figure S8: Properties of compounds (**1**, **2**, **4**, **6**, and **7**) at different pHs (1 to 12) in mixing CH_3_OH/H_2_O in a 1:1 volume ratio; Figure S9: Colocalization scatter plots correspond to the calculated Pearson’s coefficient. Ref. [49] is cited in the Supplementary Materials.
