# Supplementary material for: Spectroscopic and Biological Properties of the 3-Imino-1,8-naphthalimide Derivatives as Fluorophores for Cellular Imaging
Source: Molecules. 2023 Aug 25;28(17):6255. doi: 10.3390/molecules28176255 (PMC10488415; doi:10.3390/molecules28176255)
Supplement: Supplementary file 1 [file molecules-28-06255-s001.zip › molecules-2564672-supplementary.pdf]

Supplementary Materials

Spectroscopic and biological properties of the 3-imino-1,8-naphthalimide derivatives as fluorophores for cellular imaging

Mateusz Korzec[*,1], Sonia Kotowicz[1], Katarzyna Malarz[2], Anna Mrozek-Wilczkiewicz[2]

[1]*Institute of Chemistry, University of Silesia, 9 Szkolna Str., 40-006 Katowice, Poland*

[2]*A. Chelkowski Institute of Physics, University of Silesia in Katowice,1A 75 Pulku Piechoty Str., 41-500 Chorzow, Poland*

* corresponding author: mateusz.korzec@us.edu.pl

1. Characterization Methods

The synthesis were performed using IKA C-MAG HS7 and VWR VMS-C7 hot plates. Nuclear magnetic resonance (^{1}H and ^{13}C NMR) spectra were recorded on a Bruker AC400 spectrometer in $CDCl_3$ as solvent and TMS as the internal standard. The elementary analysis was performed using Vario EL III apparatus (Elementar, Germany). Differential Scanning UV-Vis absorption spectra were performed using an Evolution 220 UV-Visible Spectrophotometer and Hitachi U-2900 as the concentration of 10^{-4} mol/L and 10^{-5} mol/L and 1 cm quartz cell. Photoluminescence spectra (PL) in solutions were performed by using Varian Carry Eclipse Spectrometer. Quantum yields (Φ_f) measurements were performed by using the integrating sphere AvantesAvaSphere-80 (Edinburgh Instruments) and absolute method. The pH-meter CPC-511 was used to determine the pH of the MeOH/H_2O mixture (v/v; 1:1).

2. *Synthesis and characterization of compounds*

The synthesis of compounds was performed using the method described in previous works [1-4]. The characteristics of compounds 1-5 are given in the earlier [4] work, while the new compounds (6-8) are given below.

2.1. General procedure synthesis of 3-nitro-1,8-naphthalimides

4 mmol of 3-nitro-1,8-naphthalicanhydride (0.972 g) and 25 mL of EtOH were introduced to a round-bottomed flask. The resulting suspension was heated to reflux, and 8 mmol of a suitable liquid amine (4-methylobenzylamine, 4-fluorobenzylamine) was added. After 2 h of reaction, the mixture was cooled, filtered off, washed with ethanol and air-dried. The compound was used in the next step without purification

2.2. General procedure synthesis of 3-amino-1,8-naphthalimides

3.5 mmol of N-substituted-3-nitro-1,8-naphthalimide, 1.5 mL of hydrazine and 40 mg of Pd/C in 35 mL of EtOH were placed in a flask. The reduction was carried out for 4 h under a nitrogen atmosphere. Then, the solvent was evaporated and the obtained mixture was purified by extraction with dichloromethane and water.

2.3. General procedure synthesis of 3-imino-1,8-naphthalimides

1 mmol of the corresponding amine, 1 mmol of the 2,2′-Bithiophene-5-carboxaldehyde or benzothiazole-2-carbaldehyde, 10 mL of EtOH and three drops trifluoroacetic acid were introduced into the reaction. Next, the vial was closed with a septum. The reaction was carried out for 2 h in an ultrasonic bath, after which the mixture was put into the freezer, and then filtered and washed with ethanol. The product was crystallized from ethyl acetate.

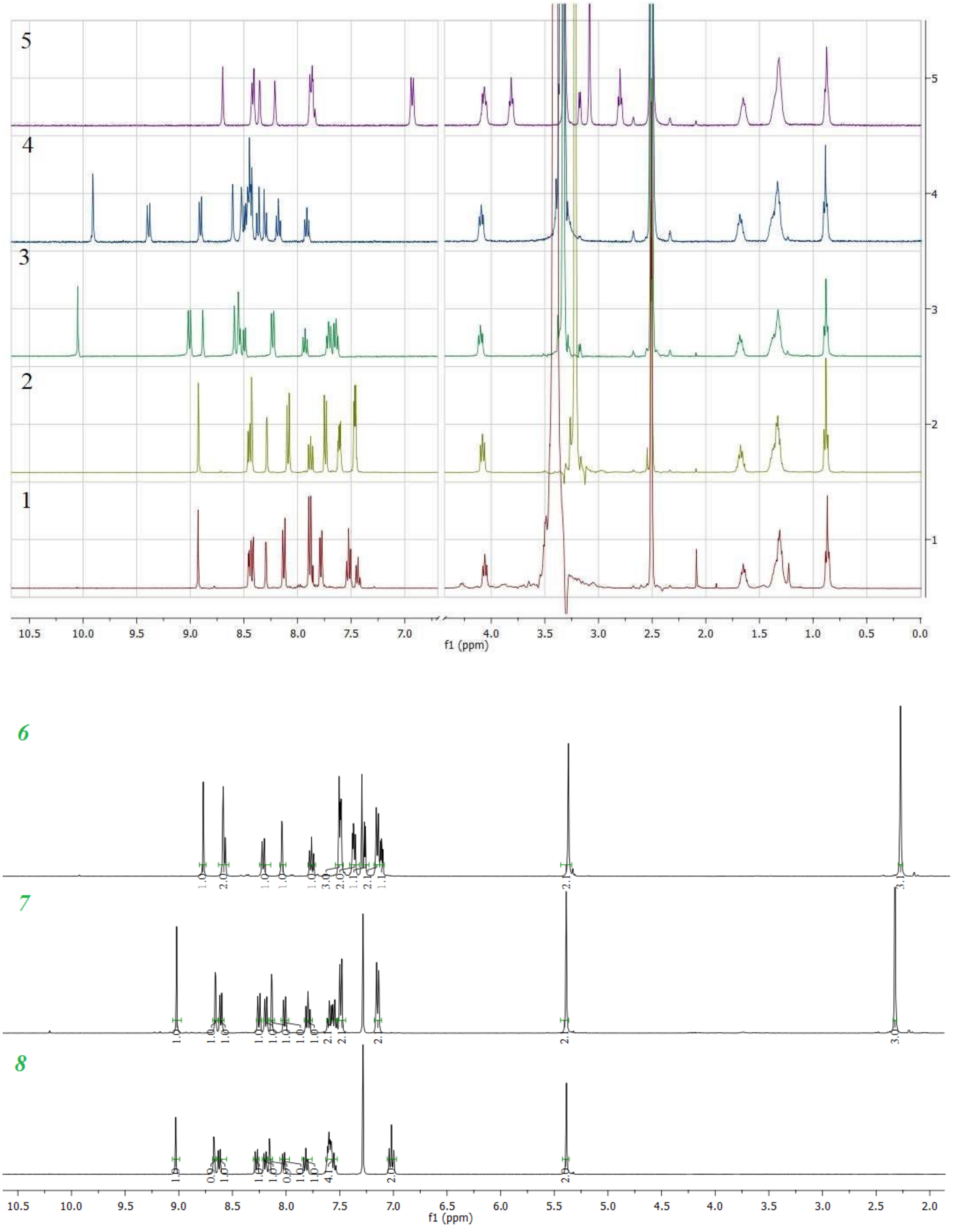

Figure S1.^{1}H NMR in DMSO-d_6 for compounds 1-5 and in $CDCl_3$ for compounds 6 -8.

Compound 1

^{1}**H NMR** ***(400 MHz, DMSO-d$_6$, δ, ppm):*** 8.93 (s, 1H, -CH=N-); 8.47 – 8.39 (m, 3H, -CH); 8.30 (d, *J*=2.0 Hz, 1H, -CH); 8.13 (d, *J*=8.3 Hz, 2H, -CH); 7.91 – 7.85 (m, 3H, -CH); 7.78 (d, *J*=7.3 Hz, 2H, -CH); 7.52 (t, *J*=7.5 Hz, 2H, -CH); 7.44 (t, *J*=7.3 Hz, 1H, -CH); 4.06 (t, *J*=7.3 Hz, 2H, -N-CH_2-); 1.69 – 1.59 (m, 2H, -CH_2-); 1.40 – 1.25 (m, 6H, -CH_2-); 0.87 (t, *J*=6.9 Hz, 3H, -CH_3). ^{13}**C NMR** ***(101 MHz, DMSO-d$_6$, δ, ppm):*** The product was insufficiently soluble for analysis. ^{1}**H NMR** ***(400 MHz, CDCl$_3$, δ, ppm):*** 8.71 (s, 1H, -CH=N-); 8.55 (d, *J*=5.8 Hz, 2H, -CH); 8.21 (d, *J*=8.2 Hz, 1H-CH); 8.06 (d, *J*=7.9 Hz, 2H, -CH); 8.00 (s, 1H, -CH); 7.77 (d, *J*=7.9 Hz, 3H, -CH); 7.69 (d, *J*=7.7 Hz, 2H, -CH); 7.51 (t, *J*=7.3 Hz, 2H, -CH); 7.43 (t, *J*=7.2 Hz, 1H, -CH); 7.28 (s, 1H,-CH); 4.20 – 4.15 (m, 2H, -N-CH_2-); 1.85 – 1.70 (m, 2H, -CH_2-); 1.53 – 1.29 (m, 6H, -CH_2-); 0.96 – 0.95 (m, 3H, -CH_3). ^{13}**C NMR** ***(101 MHz, CDCl$_3$, δ, ppm):*** 164.1; 164.0; 161.9; 150.0; 144.8; 140.1; 134.7; 133.6; 132.0; 130.3; 129.7; 129.0; 128.1; 127.0; 127.6; 127.4; 127.4; 127.2; 126.5; 125.1; 124.7; 123.9; 122.8; 113.9; 40.6; 31.6; 28.1; 26.8; 22.6; 14.1. **Anal. Calcd** for $C_{31}H_{28}N_2O_2$ (460.56 g/mol): C, 80.84; H, 6.13; N, 6.08; found: C, 80.79; H, 6.11; N, 6.08.

Compound 2

^{1}**H NMR** ***(400 MHz, DMSO-d$_6$, δ, ppm):*** 8.95 (s, 1H, -CH=N-); 8.48 – 8.40 (m, 3H, -CH); 8.33 (d, *J*=2.0 Hz, 1H, -CH); 8.10 (d, *J*=8.2 Hz, 2H, -CH); 7.89 (t, *J*=7.7 Hz, 1H, -CH); 7.76 (d, *J*=8.2 Hz, 2H, -CH); 7.66 – 7.56 (m, 2H, -CH); 7.53 – 7.41 (m, 3H, -CH); 4.12 – 4.01 (m, 2H, -N-CH_2-); 1.74 – 1.58 (m, 2H, -CH_2-); 1.43 – 1.22 (m, 6H, -CH_2-); 0.88 (t, *J*=6.8 Hz, 3H, -CH_3). ^{13}**C NMR** ***(101 MHz, DMSO-d$_6$, δ, ppm):*** 163.3; 163.7; 162.7; 150.2; 136.2; 134.6; 132.9; 132.3; 131.9; 130.4; 129.6; 129.3; 128.2; 126.4; 126.2; 125.5; 124.9; 123.8; 122.7; 122.5; 92.4; 89.5; 31.4; 27.9; 26.6; 22.4; 14.22. **Anal. Calcd** for $C_{33}H_{28}N_2O_2$ (484.59 g/mol): C, 81.79; H, 5.82; N, 5.78; found: C, 81.32; H, 5.68; N, 5.93.

Compound 3

^{1}**H NMR *(400 MHz, DMSO-d$_6$, δ, ppm):*** 10.05 (s, 1H, -CH=N-); 9.01 (d, *J*=8.7Hz, 2H, -CH); 8.88 (s, 1H, -CH); 8.56 (m, 3H, -CH); 8.50 (d, *J*=7.2Hz, 1H, -CH); 8.23 (d, *J*=8.3Hz, 2H, -CH); 7.93 (t, *J*=7.8Hz, 1H, - CH); 7.93 – 7.98 (m, 2H, -CH), 7.67 – 7.61 (m, 2H, -CH); 4.16 – 4.02 (m, 2H, -N-CH$_2$-); 1.76 – 1.59 (m, 2H, -CH$_2$-); 1.44 – 1.20 (m, 6H, -CH$_2$-); 0.88 (t, *J* =6.9Hz, 3H, -CH$_3$). ^{13}**C NMR *(101 MHz, DMSO-d$_6$, δ, ppm):*** 163.7; 163.6; 162.1; 155.6; 150.8; 140.5; 137.9; 134.6; 132.9; 130.8; 130.3; 130.1; 129.4; 129.2; 129.1; 128.5; 128.2; 126.9; 126.1; 124.9; 124.3; 123.7; 122.5; 31.4; 27.9; 26.6; 22.4; 14.4. **Anal. Calcd** for $C_{33}H_{28}N_2O_2$ (484.59 g/mol): C(81.79%) H(5.82%) N(5.78%); found: C(81.79%) H(5.81%) N(5.81%).

Compound 4

^{1}**H NMR *(400 MHz, DMSO-d$_6$, δ, ppm):*** 9.91 (s, 1H, -CH=N-); 9.39 (d, *J*=9.4Hz, 1H, -CH); 8.91 (d, *J*=8.1Hz, 1H, -CH); 8.61 (m, 1H, -CH); 8.53 – 8.42 (m, 7H, -CH); 8.37 (d, *J*=8.9Hz, 1H, -CH); 8.30 (d, *J*=8.9Hz, 1H, -CH); 8.18 (t, *J*=7.7Hz, 1H, -CH); 7.91 (t, *J*=7.8Hz, 1H, -CH); 4.15 – 4.01 (m, 2H, -N-CH$_2$-); 1.75 – 1.62 (m, 2H, -CH$_2$-); 1.42 – 1.28 (m, 6H, -CH$_2$-); 0.88 (t, *J*=6,9Hz, 3H, -CH$_3$). ^{13}**C NMR *(101 MHz, DMSO-d$_6$ or CDCl$_3$, δ, ppm):*** The product was insufficiently soluble for analysis. **Anal. Calcd** for $C_{35}H_{28}N_2O_2$ (508.61 g/mol): C(82.65%) H(5.55%) N(5.51%); found: C(82.03%) H(5.52%) N(5.42%).

Compound 5

^{1}**H NMR *(400 MHz, DMSO-d$_6$, δ, ppm):*** 8.70 (s, 1H, -CH=N-); 8.42 (d, *J*=7.1Hz, 2H, -CH); 8.35 (s, 1H, -CH); 8.21 (s, 1H, -CH); 7.91 – 7.81 (m, 3H, -CH); 6.93 (d, *J*=8.8Hz, 2H, -CH); 4.13 – 4.02 (m, 2H, -N-CH$_2$-); 3.81 (t, *J*=6.5Hz, 2H, -N-CH$_2$-); 3.08 (s, 3H, -N-CH$_3$); 2.80 (t, *J*=6.6Hz, 2H, -CH$_2$-); 1.74 – 1.57 (m, 2H,-CH$_2$-); 1.41 – 1.26 (m, 6H, -CH$_2$-); 0.88 (t, *J*=6.6Hz, 3H, -CH$_3$). ^{13}**C NMR *(101 MHz, DMSO-d$_6$, δ, ppm):*** 163.9; 163.8; 162.5; 151.6; 151.4; 134.3; 133.1; 131.3; 129.8; 127.9; 125.9; 125.9; 124.9; 124.1; 123.6; 122.6; 119.7; 112.36; 47.9; 38.4;

31.3; 27.9; 26.6; 22.4; 15.6; 14.2. **Anal. Calcd** for $C_{29}H_{30}N_4O_2$ (466.57 g/mol): C(74.65%) H(6.48%) N(12.01%); found: C(74.33%) H(6.45%) N(11.94%).

Compound 6

^{1}H NMR (400 MHz, $CDCl_3$, δ, ppm): δ 8.74 (s, 1H), 8.55 (t, 2H), 8.19 (d, *J* = 8.1 Hz, 1H), 8.02 (d, 1H), 7.75 (t, 1H), 7.51 – 7.45 (m, 3H), 7.38 – 7.34 (m, 2H), 7.25 (d, 1H), 7.20 – 7.08 (m, 3H), 5.38 (s, 2H), 2.32 (s, 3H). ***^{13}C NMR (101 MHz, $CDCl_3$, δ, ppm):*** δ 164.12, 164.03, 154.36, 149.59, 143.32, 140.60, 137.18, 136.73, 134.35, 133.78, 132.64, 130.48, 129.12, 129.06, 128.24, 127.45, 126.53, 126.16, 125.63, 125.34, 124.86, 124.16, 123.76, 122.71, 43.37, 21.13.***Anal. Calcd for*** $C_{29}H_{20}N_2O_2S_2$ (492.61 g/mol): C(70.71%) H(4.09%) N(5.69%); found: C (71.05%) H(4.20%) N (5.62%).

Compound 7

^{1}H NMR (400 MHz, $CDCl_3$, δ, ppm):δ 9.02 (s, 1H), 8.66 (d, 1H), 8.61 (d, *J* = 7.3 Hz, 1H), 8.25 (d, *J* = 8.2 Hz, 1H), 8.17 (t, 1H), 8.13 (d, 1H), 8.01 (d, *J* = 7.7 Hz, 1H), 7.80 (t, 1H), 7.66 – 7.52 (m, 2H), 7.49 (d, *J* = 8.0 Hz, 2H), 7.15 (d, *J* = 7.9 Hz, 2H), 5.39 (s, 2H), 2.32 (s, 3H). ***^{13}C NMR (101 MHz, $CDCl_3$, δ, ppm):*** δ 166.48, 163.96, 163.75, 155.60, 153.98, 148.12, 137.24, 135.67, 134.22, 134.11, 132.49, 131.25, 129.14, 129.06, 127.79, 127.40, 126.81, 125.82, 125.79, 124.88, 124.62, 124.15, 122.87, 122.20, 43.34, 21.12. ***Anal. Calcd for*** $C_{28}H_{19}N_3O_2S$ (461.53 g/mol): C(72.87%) H(4.15%) N(9.10%); found: C (72.75%) H(4.32%) N (9.14%).

Compound 8

^{1}H NMR (400 MHz, $CDCl_3$, δ, ppm):δ 9.03 (s, 1H), 8.67 (d, 1H), 8.62 (d, *J* = 7.2 Hz, 1H), 8.28 (d, *J* = 8.2 Hz, 1H), 8.20 (d, *J* = 8.1 Hz, 1H), 8.15 (d, 1H), 8.02 (d, *J* = 7.7 Hz, 1H), 7.82 (t, 1H), 7.66 – 7.49 (m, 4H), 7.02 (t, 2H), 5.39 (s, 2H). ***^{13}C NMR (101 MHz, $CDCl_3$, δ, ppm):*** δ 166.42, 163.95, 163.74, 163.48, 155.68, 153.98, 148.20, 135.68, 134.25, 132.54, 131.32,

131.10, 131.02, 127.83, 127.33, 126.83, 125.84, 125.01, 124.64, 124.02, 122.74, 122.20, 115.38, 115.17, 42.96. ***Anal. Calcd for*** $C_{27}H_{16}FN_3O_2S$ (465.50 g/mol): C(69.66%) H(3.46%) N(9.03%); found: C (70.15%) H(3.27%) N (8.92%).

3.Optical properties testing

3.1. Sample preparation by direct dissolution

The solutions were prepared by weighing each compound on an analytical balance in the amount of approx. 0.5 mg (depending on the molar mass) for each tested solvent (*dichloromethane, acetonitrile, methanol*), and then dissolving it in the test solvent (10 mL), which allowed to obtain a solution with a concentration of 100 ◎M (10^{-4} mol/dm^3). All tested compounds were dissolved in dichloromethane, compound 4 did not dissolve in acetonitrile, and compounds 3, 4, 6, 7, 8 did not dissolve in methanol. Then, 1 mL of the previously prepared solution was collected into a 10 mL volumetric flask and made up to the mark with the test solvent. Thus, solutions with a concentration of 10 ◎M (10^{-5} mol/dm^3) were obtained. Spectroscopic examinations were carried out 2 hours after the preparation of the solutions.

3.2. Sample preparation by pre-dissolving in DMSO and $CHCl_3$

The solutions were prepared by weighing each compound in the amount of approx. 0.3 mg (depending on the molar mass) on an analytical balance, and then initially dissolving it in DMSO and analogous samples dissolved in $CHCl_3$, which allowed to obtain a solution with a concentration of 1 mM (10^{-3} mol/dm^3). All compounds dissolved in both solvents. Next, 0.1 mL of the previously prepared solution was collected into a 10 mL volumetric flask and made up to the mark with the test solvent (dichloromethane, acetonitrile, methanol). Thus, solutions with a concentration of 10

◎M (10^{-5} mol/dm^3) were obtained. Spectroscopic examinations were carried out 2 hours after the preparation of the solutions.

3.3. Preparation of samples for aggregation tests

The solutions were prepared by weighing each compound in the amount of approx. 0.6 mg (depending on the molar mass) on an analytical balance, and then initially dissolving it in DMSO, which allowed to obtain a solution with a concentration of 1 mM (10^{-3} mol/dm^3). For each tested compound, 10 mL (in a volumetric flask) of appropriate MeOH/H_2O systems were prepared with increasing water content, corresponding to its percentage volume, i.e .: fw: 0, 10, 20, 30, 40, 50, 60, 70, 80, 90. Next, 0.1 mL of the solution of compound in DMSO was added to the prepared MeOH/H_2O systems and mixed. Thus, solutions with a concentration of 10 ◎M (10-5 mol/dm3) were obtained. Spectroscopic examinations were carried out 2 hours after the preparation of the solutions.

3.4. Preparation of samples for PET inhibition studies

The solutions were prepared by weighing each compound in the amount of approx. 0.6 mg (depending on the molar mass) on an analytical balance, and then initially dissolving it in DMSO, which allowed to obtain a solution with a concentration of 1 mM (10^{-3} mol/dm^3). A trifluoroacetic acid (TFA) solution was prepared by weighing out 2.85 mg on an analytical balance and then quantitatively transferring it to a volumetric flask with a capacity of 25 mL with test solvent (separately in acetonitrile and in methanol). Then the flasks were made up to the mark with the solvent, thus obtaining a solution with a concentration of 100 ◎M (10^{-3} mol/dm^3). Subsequently, sets of 7 volumetric flasks with a volume of 10 mL were prepared for each compound. Each flask was made up to half with the solvent, and then the previously prepared TFA solution was added in the following amounts: 0, 0.01, 0.03, 0.08, 0.1, 0.3 and 1 mL, respectively. Then 0.1 mL of the test compound was introduced into each flask. The flasks were made up to the mark

with solvent and mixed. Spectroscopic examinations were carried out 2 hours after the preparation of the solutions.

4. *Biological studies*

4.1. Cell culture

The human breast cancer cell line (MCF-7), the human colorectal carcinoma cell line (HCT 116), and the human glioblastoma cell line (U-251) were purchased from the ATCC. The normal human dermal fibroblasts (NHDF) was obtained from PromoCell. The cell lines were cultured in Dulbecco's modified Eagle's medium/Nutrient Mixture F-12 (DMEM) with 10% heat-inactivated fetal bovine serum – FBS (for cancer cells) or 15% non-inactivated FBS (for NHDF) and a mixture of antibiotics: streptomycin and penicillin (1% v/v) (all reagents from Sigma-Aldrich). All of cell lines were grown under standard conditions at 37°C and in a humidified atmosphere at 5% CO_2.

4.2. Cytotoxicity studies

The cell lines were seeded at a density of 5 000 cells per well (for MCF-7, HCT 116, U-251) and 4 000 cells per well (for NHDF) into 96-well clear plates (Nunc) and incubated under standard conditions for one day. After 24h, the complete DMEM was exchanged for solutions of tested compounds at concentrations ranging from 1 to 25 μM. After a 72 h incubation, the cytotoxicity assay - CellTiter 96® AQ_{ueous} One Solution Cell Proliferation Assay – MTS (Promega) was performed as standard protocol. Briefly, the solutions of the tested compounds were removed, and 100 μL DMEM (without FBS and phenol red - PF) and 20 μL of MTS reagent were added and incubated for 1 h at 37°C. After this time, the optical densities of wells with controls (untreated cells) and tested compounds were measured at 490 nm using a multi-plate reader -Varioskan LUX (ThermoScientific). The obtained absorbance values are expressed as the percentage of

the control and were calculated as the inhibitory concentration (IC_{50}) using GraphPad Prism 9. Each individual compound was tested in triplicate in a single experiment with each experiment being performed three times.

4.3. Cellular staining

Before cellular staining experiments, MCF-7 cells were seeded onto coverslips at a density of 140,000 cells per slide and incubated at 37 °C for 48 h. Then, the DMEM was removed, and solutions of the tested compounds at a concentration of 25μM were added and further incubated for 2 h. Then, the cells were washed three times with PBS and mounted with DMEM without FBS or phenol red. The cellular staining results were immediately observed after excitation at 365 nm (DAPI filter), and 470 nm (GFP filter) LED illumination (25% of power) using a Zeiss Axio Observer.Z1 inverted fluorescence microscope equipped with an AxioCamMRm camera.

4.4. Subcellular localization

MCF-7 cells were seeded in the same manner as described above. Then, the DMEM was removed, and the solution of the compound 5 (25 μM) was added and further incubated for 2 h. After this time, the cells were washed twice with PBS and the medium (without FBS and PF) that containedMitoTracker Orange CMTMRos (100 nM),ERTracker Red BODIPY (1 μM), or LysoTracker Red DND-99 (500 nM) (all from Molecular Probes) were added and incubated for 30 min or 1 h at 37°C. Then, MCF-7 cells were washed three times with PBS and mounted with DMEM without FBS or phenol red. Cellular imaging was performed using the Zeiss Axio Observer.Z1 inverted fluorescence microscopeunder an appropriate filter for compound5 and dyes used and a 40× objective. The fluorescence images were processed using ImageJ software 1.41 (Wayne Rasband, National Institutes of Health, Bethesda, MD, USA).The Pearson’s

coefficient, which was used to show the colocalization of compound 5 with specific-organelle dyes, was calculated using the Image J plugin "JACoP" [5].

5. *Optical properties*

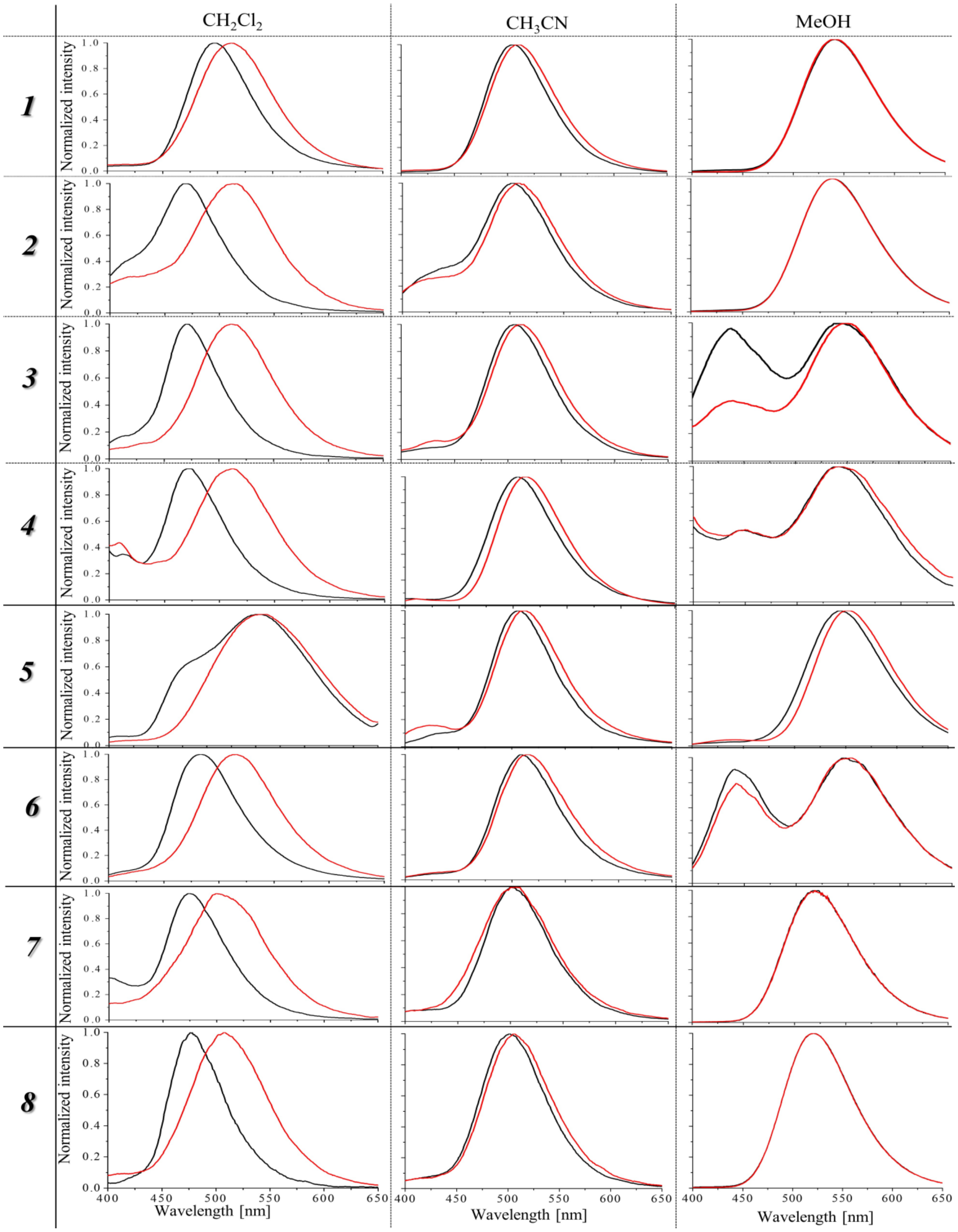

Figure S2. Emission spectra of the tested compounds depending on the sample preparation method, i.e. initial dissolution in DMSO (***red line***) or $CHCl_3$ (***black line***). Measurements were made in three solvents: CH_2Cl_2, CH_3OH, CH_3CN.

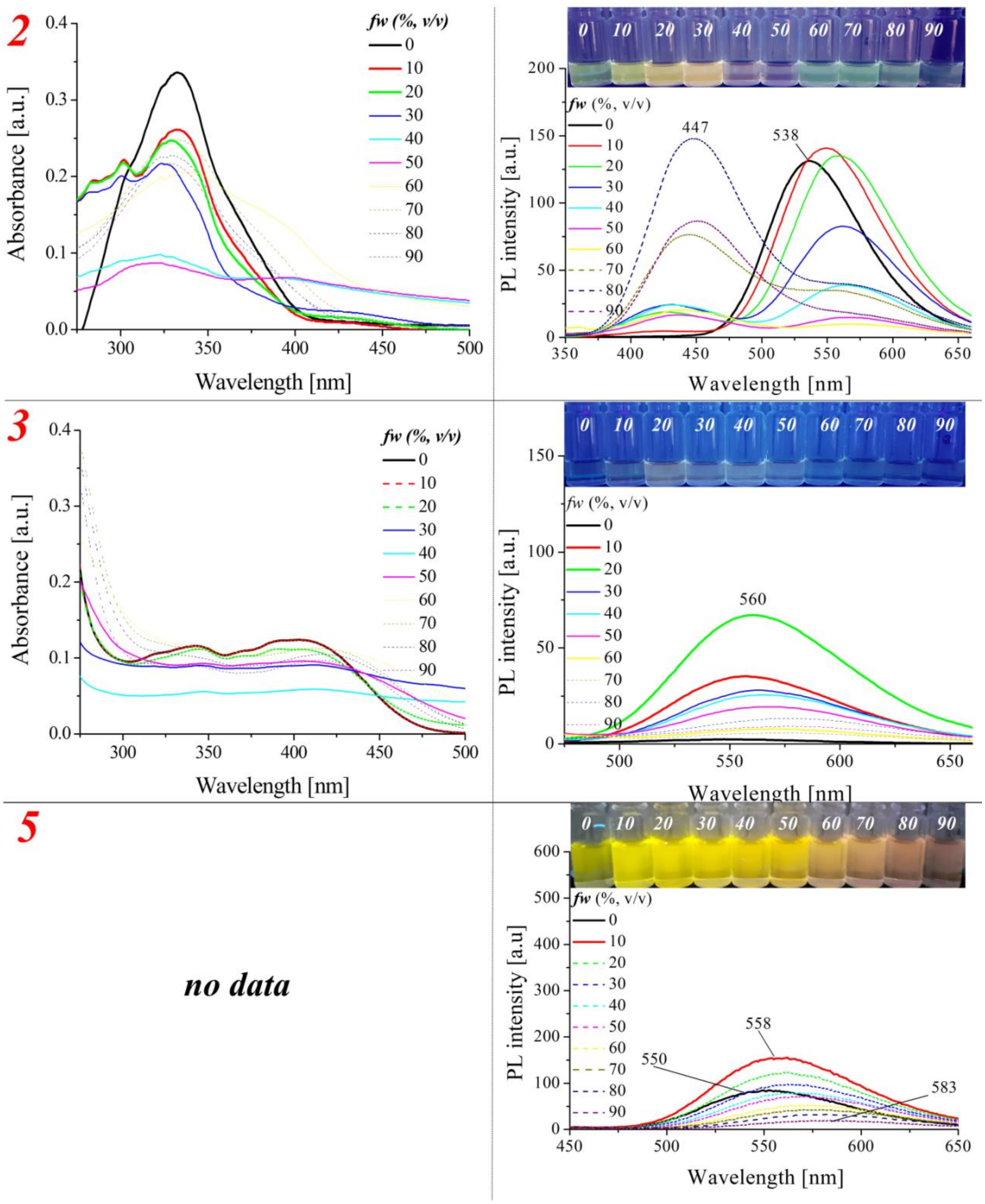

Figure S3. Absorbance and photoluminescence (PL) properties in a binary mixture of MeOH/H_2O with an increasing water content (fw) for imines (2,3,5). Photographs were taken under 365 nm UV irradiation from a hand-held UV lamp.

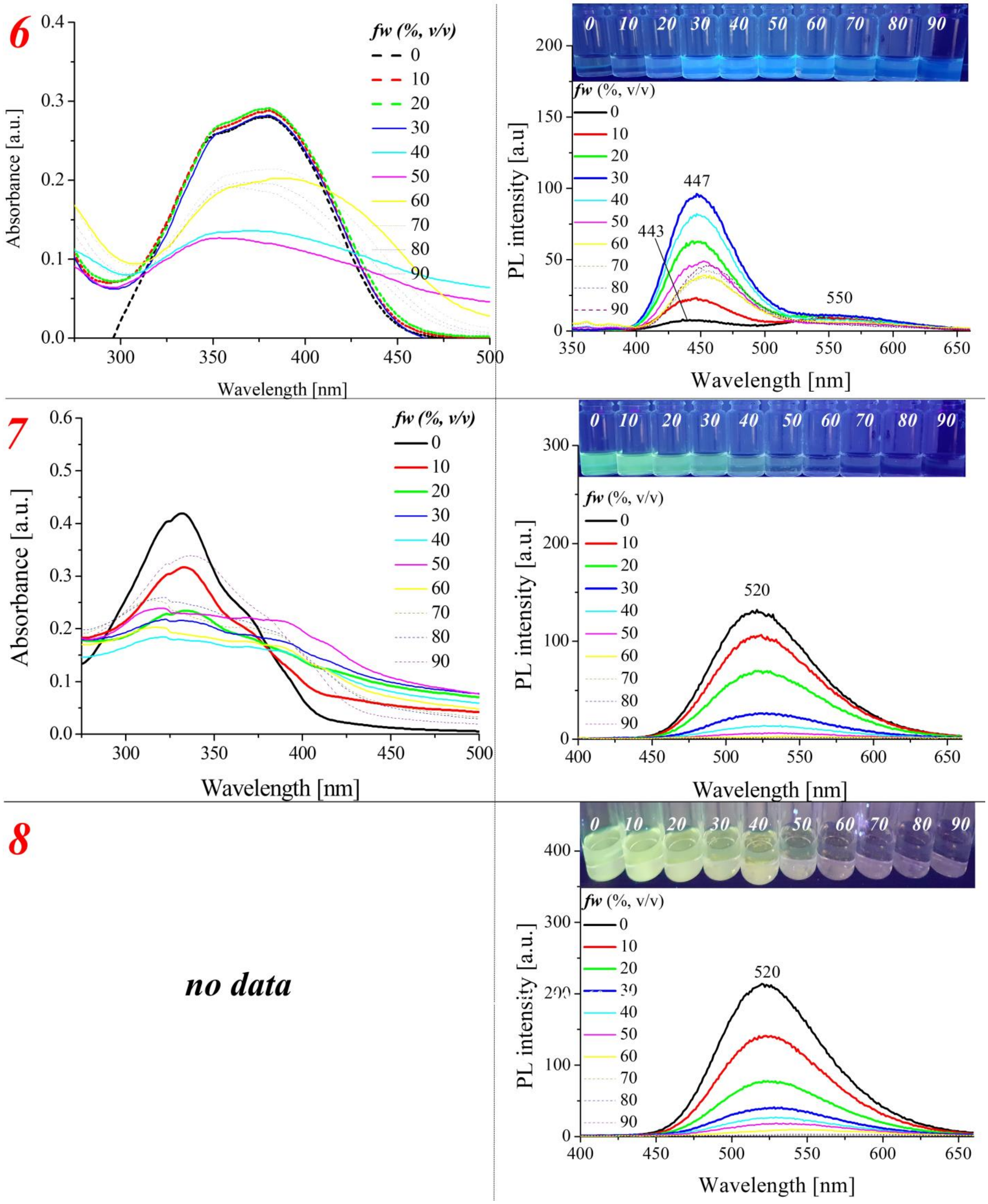

Figure S4. Absorbance and photoluminescence (PL) properties in a binary mixture of MeOH/H_2O with an increasing water content (fw) for imines (6,7,8). Photographs were taken under 365 nm UV irradiation from a hand-held UV lamp.

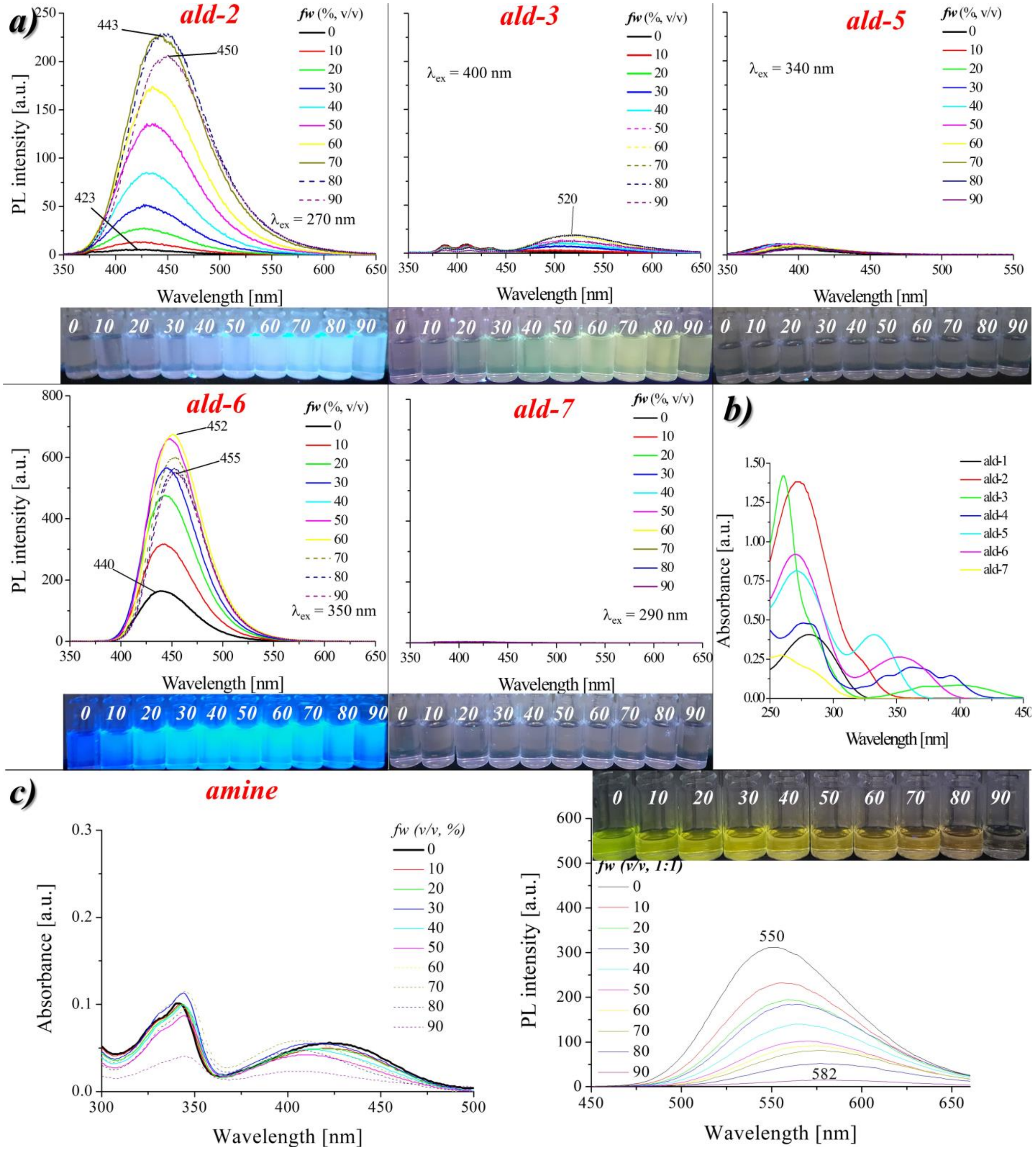

Figure S5. *a)*The photoluminescence (PL) properties in a binary mixture of MeOH/H_2O with an increasing water content (f_w) for the corresponding aldehydes (ald-2,3,5,6,7). Photographs were taken under 365 nm UV irradiation from a hand-held UV lamp, *b)* UV-VIS spectra for tested aldehydes in methanol at a concentration of 10 ◎M. and ***c)***absorption and emission properties in a binary mixture of MeOH/H_2O with an increasing water content (f_w) for the 3-amino-N-hexyl-1,8-naphthalimide.

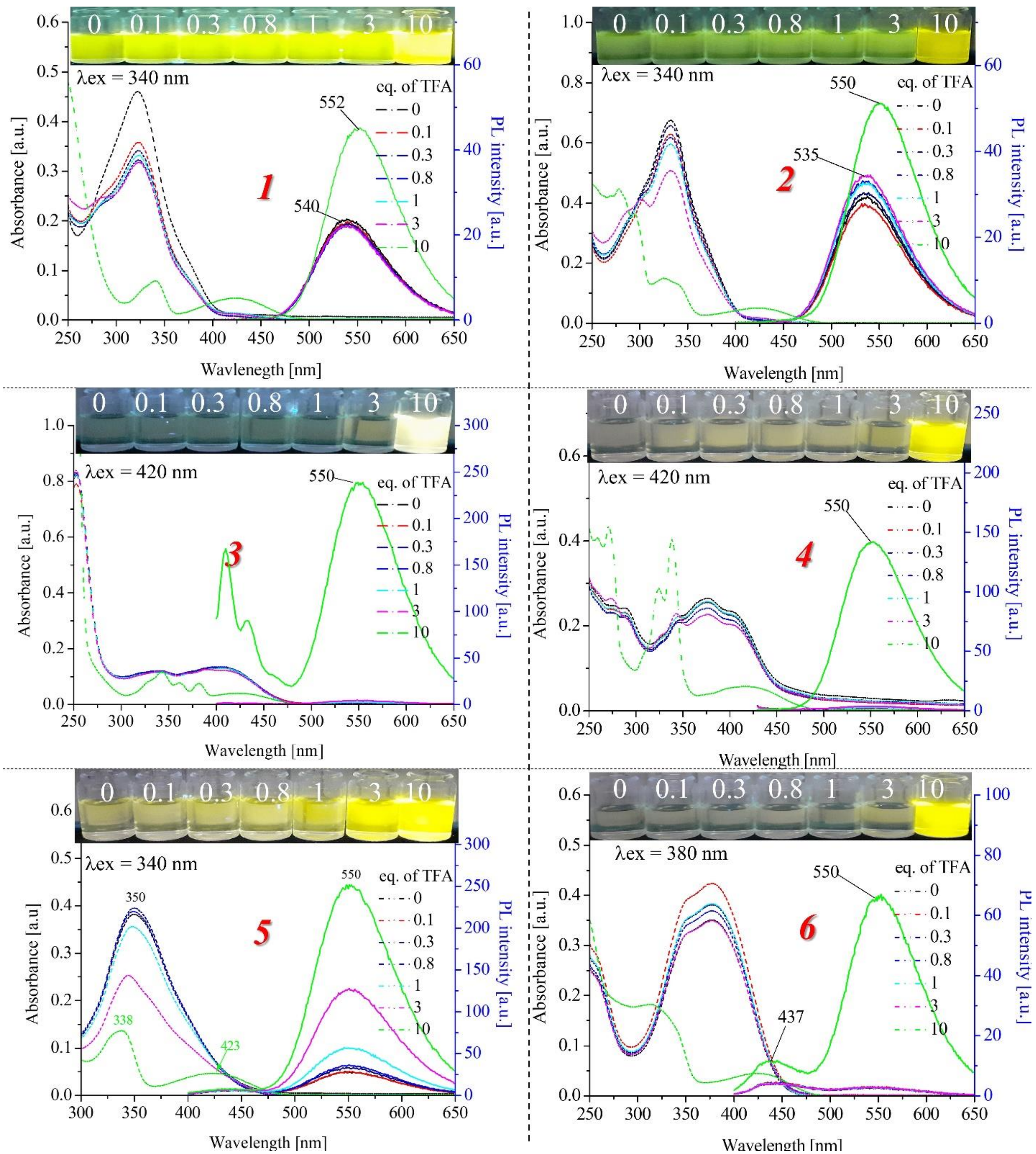

Figure S6. Effect of TFA on photoluminescence (PL) properties of imines in methanol. Photographs were taken under 366 nm UV irradiation from a hand-held UV lamp.

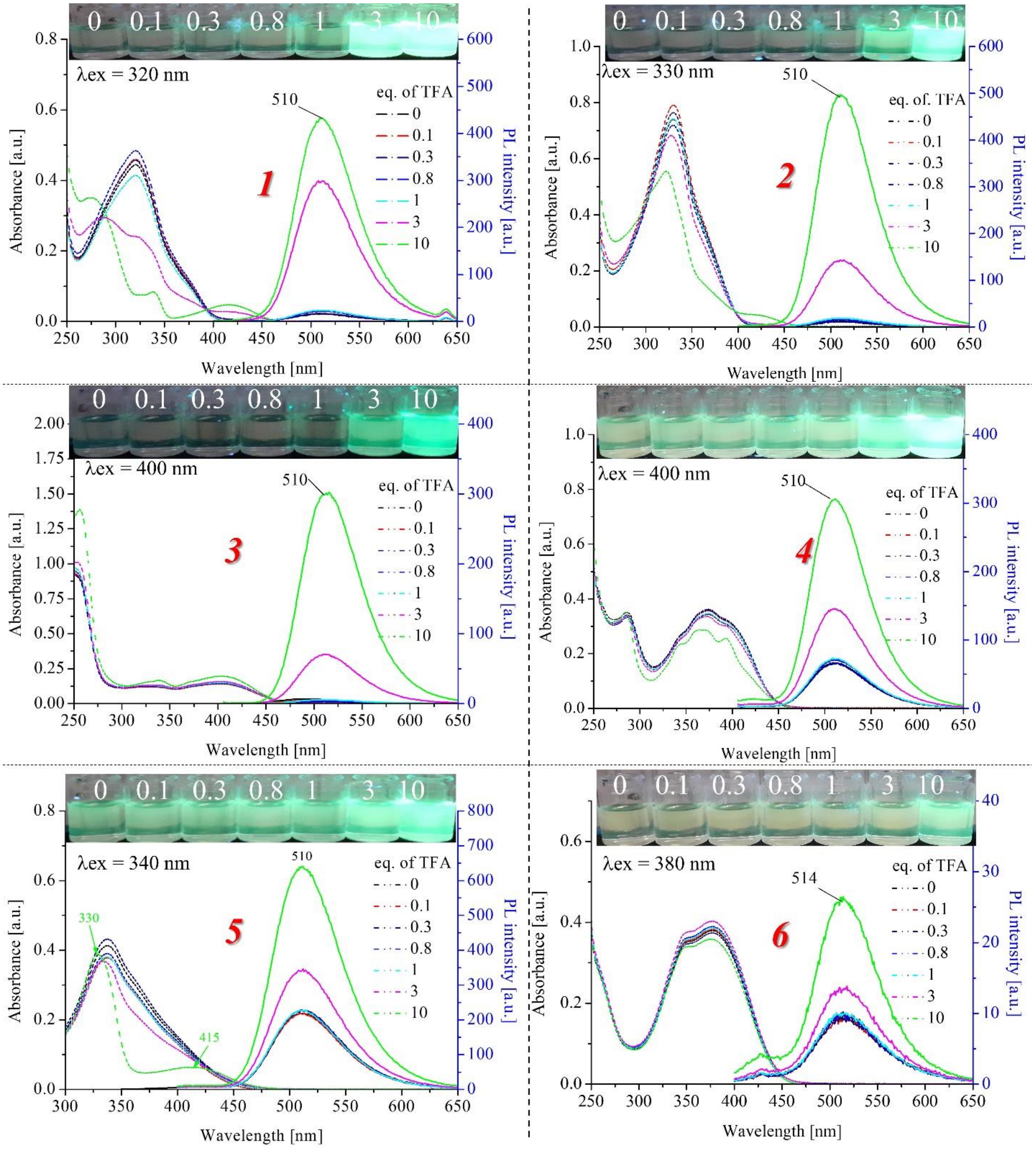

Figure S7. Effect of TFA on photoluminescence (PL) properties of imines in acetonitrile. Photographs were taken under 366 nm UV irradiation from a hand-held UV lamp.

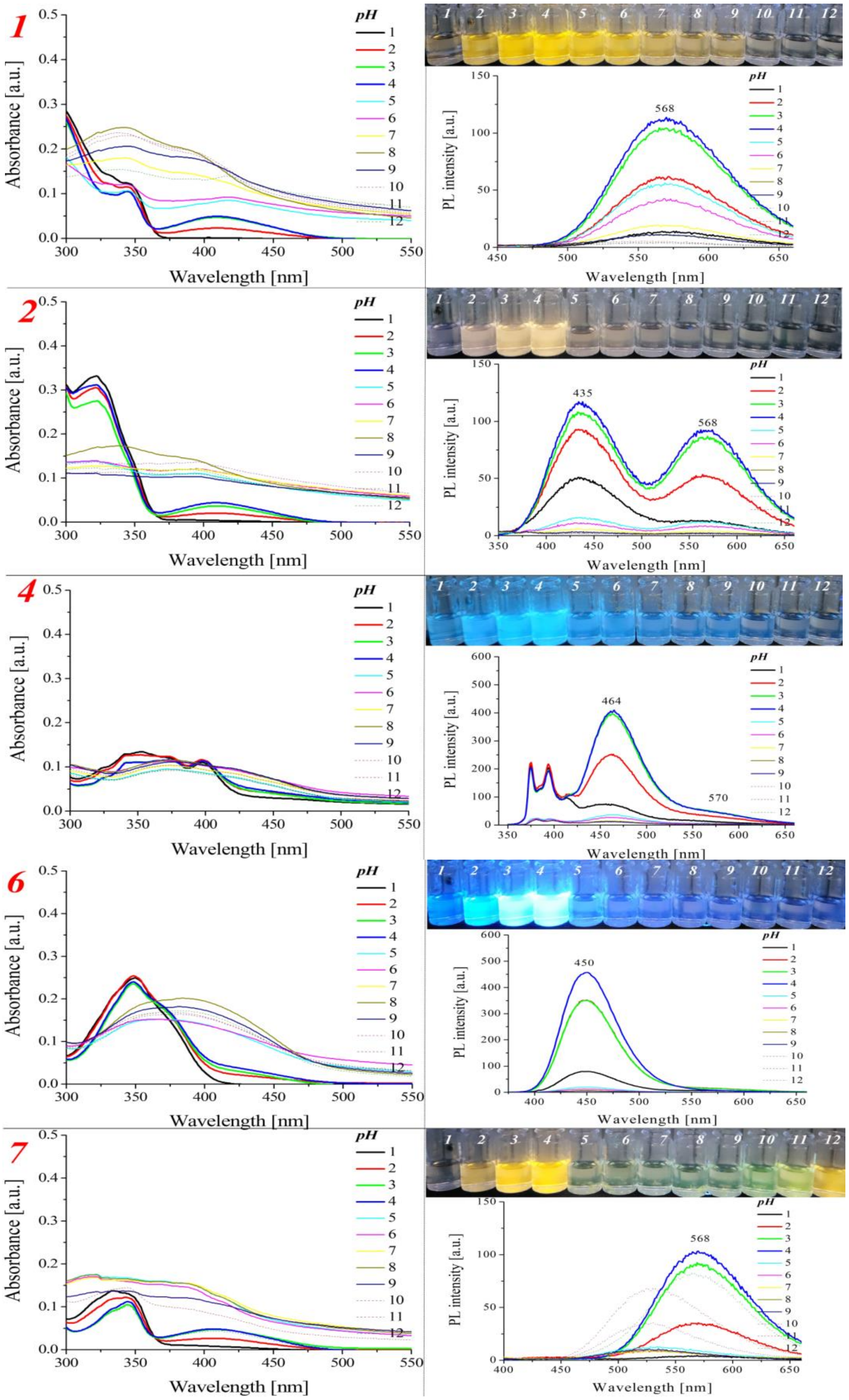

Figure S8. Properties of compounds (1,2,4,6,7) at different pH (1 to 12) in mixing CH_3OH/H_2O in a 1:1 volume ratio. The concentration of the compound is 10 ◎M. Photographs were taken under 366 nm UV irradiation from a hand-held UV lamp.

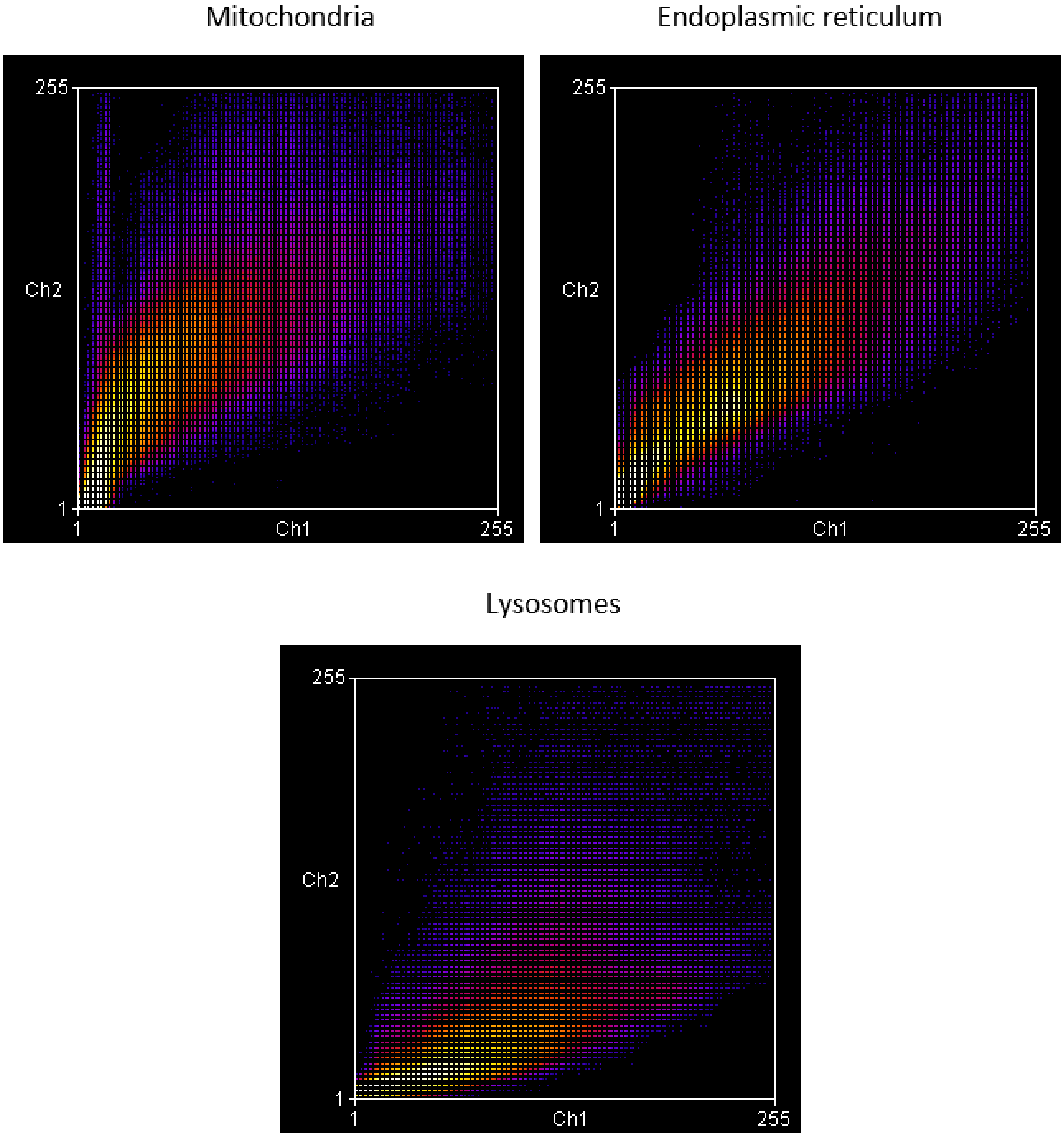

Figure S9. Colocalization scatter plots correspond to calculated Pearson's coefficient. Plots were generated from ImageJ software.

Reference

[1] M. Korzec S. Kotowicz, R. Rzycka-Korzec, E. Schab-Balcerzak, J.G. Małecki, M. Czichy, M. Łapkowski.Novel ⊚-ketoenamines versus azomethines for organic electronics: characterization of optical and electrochemical properties supported by theoretical studies,*J Mater Sci.* 55, 2020, 3812–3832.

[2] M. Korzec K. Malarz, A. Mrozek-Wilczkiewicz, R. Rzycka-Korzec, E. Schab-Balcerzak, J. Polański.Live cell imaging by 3-imino-(2-phenol)-1,8-naphthalimides: The effect of ex vivo hydrolysis. *Spectrochimica Acta Part A: Molecular and Biomolecular Spectroscopy,* 238, 2020, 118442.

[3] S. Kotowicz M. Korzec, K. Malarz, A. Krystkowska, A. Mrozek-Wilczkiewicz, S. Golba, M. Siwy, S. Maćkowski, E. Schab-Balcerzak. Luminescence and Electrochemical Activity of New Unsymmetrical 3-Imino-1,8-naphthalimide Derivatives,*Materials* . 14, 2021, 5504.

[4] S. Kotowicz M. Korzec, J.G. Małecki, M. Siwy, S. Maćkowski, E. Schab-Balcerzak. Six New Unsymmetrical Imino-1,8-naphthalimide Derivatives,*Materials.* 15, 2022, 7043.

[5] S. Bolte, F. P. Cordelières, A guided tour into subcellular colocalization analysis in light microscopy, *Journal of Microscopy*, 224 (2006) 213-232.
